# Enterovesical Fistula Caused by a Toothpick

**DOI:** 10.1155/2015/902673

**Published:** 2015-03-08

**Authors:** Flavia Tombolini, Vito Lacetera, Giovanni Muzzonigro

**Affiliations:** Department of Urology, Regional Hospital of Marche Region “Ospedali Riuniti Umberto I, Lancisi, Salesi”, Ancona, Italy

## Abstract

We present a case of enterovesical fistula caused by an accidental ingestion of a foreign body. A 23-year-old man presented to our hospital with pneumaturia, fecaluria, and abdominal pain but no recent possible causes of enterovesical fistula at anamnesis. Cystoscopy, cystography, and also colonoscopy were not able to detect the fistulous tract. Computer tomography (CT) revealed a fistula between bladder and bowels caused by a toothpick accidentally swallowed 2 years earlier. We tried to remove the foreign body endoscopically by cystoscopy and colonoscopy but with no success. The failure of endoscopic procedures required a surgical treatment. The patient underwent laparoscopic segmental resection of the sigmoid colon to remove the fistulous tract and the foreign body. The cystography revealed no external leakage of contrast from the bladder with complete resolution of the problem.

## 1. Introduction

Enterovesical fistula (EVF) is an abnormal communication between bladder and bowels and is most often caused by a chronic inflammatory disease, diverticulitis, or cancer proliferation with invasion of adjacent organs. Traumas or surgical injuries are rarely implicated in the genesis of EVFs [1]. Fistulae created by accidentally ingested foreign bodies are uncommon. Most of them pass through the gastrointestinal tract with no consequences, but in some cases they cause perforations, fistulae, or other complications [2]. Frequently, patients do not remember any accidental ingestion of foreign bodies [3]; therefore, physicians hardly consider foreign body ingestion as the cause of patient's symptoms. The duration of symptoms before diagnosis ranged from 1 day to 9 months from the ingestion [4]. We hereby report a case of EVF caused by a toothpick swallowed by a young patient 2 years earlier.

## 2. Case Presentation

**Figure 1:**
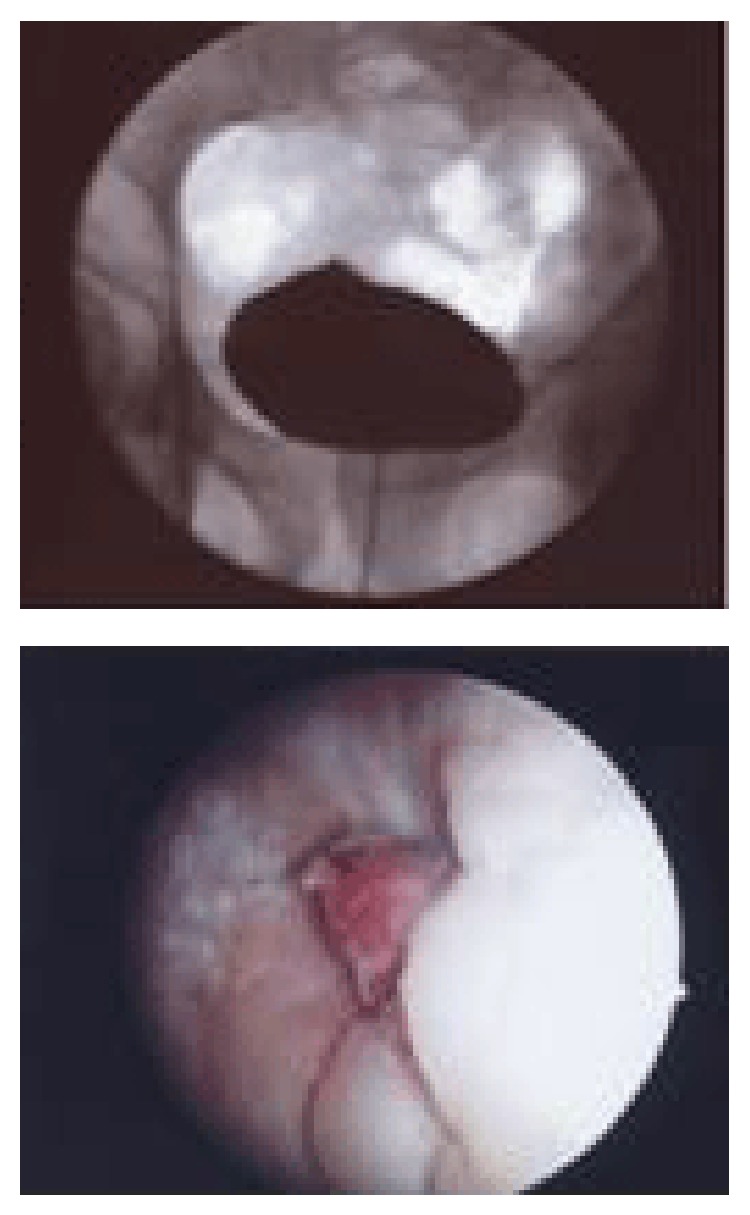
Presurgical cystography and cystoscopy. A small inflamed area is visible at cystoscopy.

**Figure 2:**
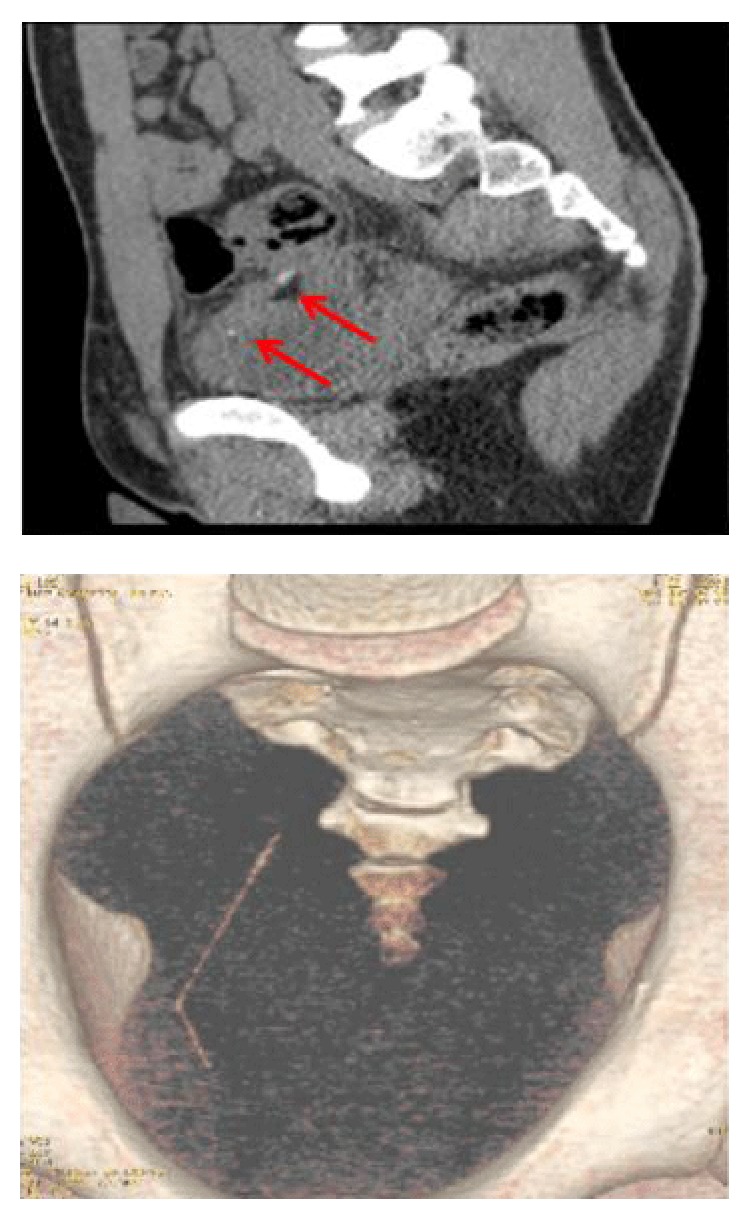
Tc scan and 3D reconstruction of pelvis. Toothpick is visible in both pictures.

A 23-year-old man presented to the Emergency Department of our hospital with a 1-month history of pneumaturia, fecaluria, and mild abdominal pain on the right inferior abdominal quadrant. He referred that, one week earlier, he experienced an episode of macroematuria with no fever and regular bowel function. He was sent to Urological Department for a consultation. His clinical history was characterized only by appendectomy and a right hernioplasty that dated back to 3 years earlier with no complication. He had no history of chronic inflammatory disease of the bowels or mental disease. Examination revealed only a right lower abdominal pain during deep palpation. An ultrasound scan of the abdomen showed a hyperechoic rim on the right wall of the bladder, but it was not considered relevant. Cystoscopy showed two relatively small inflamed areas on the right anterolateral wall of bladder. Cystography, after a refilling of 500 mL, looked normal (Figure 1). A CT of the pelvis revealed a threadlike element, probably a foreign body (length 6.5 cm and diameter 1–1.5 mm), angled in its caudal part. A tip of the foreign body was in the right anterolateral wall of bladder and the other one was in a pelvic loop of the sigmoid colon without any signs of abscess or fistula (Figure 2). After CT the patient referred that he accidentally swallowed a toothpick two years earlier with no consequent complications and then he forgot about it.

We tried to remove the foreign body endoscopically. A second cystoscopy confirmed the presence of an inflamed wall, but the foreign body was not visible inside the bladder. Gastroenterologists tried to remove the toothpick by a colonoscopy but they did not find any evidence of the foreign body in the sigmoid lumen. Even after bladder distension with methylene blue and saline solution, the colonoscopy did not revealed signs of fistula or the tip of the toothpick. The failure of endoscopic procedures required a surgical treatment. The patient underwent laparoscopic segmental resection of the sigmoid colon and transanal Knight-Griffen anastomosis to remove the fistulous tract and the foreign body. The bladder was sewed up with an interrupted polysorb 2/0 suture. At the end of the surgical operation a vesical catheter was positioned and it was removed 10 days later after a negative cystography.

## 3. Discussion

Enterovesical fistula (EFV) consists in an abnormal communication between bladder and bowel. The most common aetiology is diverticulitis, chronic inflammation diseases of the bowel (50–79% of all cases), and neoplastic disease (20% of all cases) in colon, bladder, cervix, prostate, or ovary. Fistulae between bowel and bladder can have also iatrogenic genesis [1]. EVFs caused by the presence of foreign bodies are uncommon, but they have been encountered with increasing frequency over the last decades [5]. In around 80–90% of the cases, foreign bodies accidentally ingested pass through the gastrointestinal tract uneventfully [2]. However, the incidence of perforation is higher (15–35%) if the foreign body is longer than 6.5 cm, thin and sharp [6]. The U.S. Consumer Product Safety Commission National Injury Clearinghouse from 1979 to 1982 estimated the incidence of all toothpick-related injuries to be 3.6 per 100,000 persons [7]; they account for 8-9% of the accidentally ingested foreign bodies [4, 8].

Accidental ingestion is more common in children but other predisposing factors are low QI, personality disorders, artificial dentures and orthodontic implants, rapid ingestion of food, alcohol intoxication, drug abuse, palatal insensitivity, habitual chewing of toothpick, and callousness during toothpick use [7–9].

Impaction, perforation, and obstruction generally occur at junctions, acute angulations, physical narrowing prior surgery, and congenital malformations of the gastrointestinal tract [5, 8]. Toothpicks, in particular, are indigestible and hard, with bilateral sharp ends. Because of these features, it is difficult for the toothpick to pass through the tortuous section of the gastroenteric tract or transit from a mobile portion of the bowel to a more fixed portion [4]. Therefore, the most involved sites of perforation are the duodenum and the sigmoid colon [5]. Complications include abscess, peritonitis, obstruction, and bleeding; death can occur in 20%–80% of cases [10, 11]. The migration of foreign bodies, even toothpicks, from the gastrointestinal tract to the bladder and the urinary organs rarely occurs [12].

Symptoms more frequently related to EVFs are pneumaturia, fecaluria, frequency, urgency, and dysuria. Other symptoms are soprapubic pain, haematuria, and recurrent tract infection with malodorous urines and positive urine culture tests for* Escherichia coli* or other colorectal gram negative bacteria [1]. EVFs caused by foreign bodies may complicate with enteric-vascular fistulae, peritonitis, or septic shock [5]. Some patients can complain also from intestinal symptoms. The majority of patients did not recall the swallowing of a toothpick, while only 12% remembered the ingestion of the foreign body; 21% remembered eating foods containing toothpicks without swallowing toothpick [3]. Medical errors can arise from the very first contact with the patient who does not remember the ingestion of a foreign body; the delayed diagnosis can sometimes end in a surgical emergency [9]. Another important problem in the diagnostic phase is the time between the ingestion and the symptoms manifestation. In literature symptoms can develop from 1 day to 9 months [4] and it could be difficult to relate the clinical picture to a suspected case of EVF caused by a swallowed object. In our case report the ingestion happened 2 years before the symptoms manifestation.

Imaging studies vary in sensitivity in detecting toothpick and they often prove to be inadequate [11]. At CT scan, a foreign body with a small diameter could be undetectable, also because wood has a small radiopaque density and may not be as visible as metallic objects on imaging studies [5, 7]. Transabdominal ultrasound has high sensitivity (52%–82%) in detecting wooden objects, therefore, suggesting that a foreign body could have been swallowed even if the patient does not remember it. In experimental studies, MRI showed good results and may be useful in detecting nonmetallic foreign bodies [7]. However, the definitive diagnosis of fistula caused by a toothpick has been made by endoscopy, laparoscopy, or laparotomy.

Fistulae can be treated by conservative or surgical management. Endoscopic procedures are successfully and increasingly used to remove foreign bodies [4]. An excision of the foreign body is possible when it is visible endoscopically, for example, during cystoscopy or colonoscopy. After an endoscopic management it is sufficient to carry out a medical therapy including urethral catheter to dry bladder (or, eventually, a supravesical cutaneous diversions) and antibiotics. Laparoscopic or laparotomic surgeries are preferred in case of undetectable foreign bodies or in acute abdomen. Surgical approach can be actuated in single-stage or multistage surgery. The aim of surgical management is to remove the foreign body, excise the EVF with the tract of the involved intestine, and create a reanastomosis of the bowel [1]. Surgery is important also for the resolution of complications as abscess or bleeding and also in emergency conditions as peritonitis or shocks. The bladder must be sutured with stitches. Single-stage is preferred in noncomplicated patients, but often a temporary colon or enteric diversion is necessary with a subsequent creation of anastomosis between the two extremities of bowel [1]. In postoperatory period (7–20 days) the catheterisation of bladder is useful to keep the surgical site dry [13].

## 4. Conclusion

The persistent presence of a foreign body in the pelvic area can cause inflammation of the tissue and erosion of structures with final formation of a fistulous tract. Diagnosis is not easy because a lot of patients do not remember any accidental ingestion and symptoms can start after a variable period of time from the event [3, 9]. Actually, in our case, symptoms appeared after 2 years from ingestion. In some cases, the foreign body is also not well detected by imaging studies. For example, wood toothpicks have a low radiopaque density and often CT cannot show it [5, 7]. The treatment target must be the removal of the foreign body. It may be carried out by endoscopy, when possible, or by laparoscopic (as in our case) or laparotomic surgery with a concomitant excision of the fistulous tract.
